# Determination of the DNA repair pathways utilised by acute lymphoblastic leukaemia cells following daunorubicin treatment

**DOI:** 10.1186/s13104-019-4663-8

**Published:** 2019-09-24

**Authors:** Hussain Mubarak Al-Aamri, Helen R. Irving, Terri Meehan-Andrews, Christopher Bradley

**Affiliations:** 1 Department of Pharmacy and Biomedical Sciences, La Trobe Institute for Molecular Sciences (LIMS), La Trobe University, P.O. Box 199, Bendigo, VIC 3552 Australia; 2 Oman College of Health Sciences, PO Box 293, 620 Ruwi, Oman

**Keywords:** Daunorubicin, DNA double strand breaks (DSBs), Homologous recombination pathway (HR), Non-homologous end joining (NHEJ) pathway

## Abstract

**Objective:**

DNA double strand breaks (DNA-DSBs) are among the most lethal DNA lesions leading to genomic instability and repaired by either homologous recombination (HR) or the non-homologous end joining (NHEJ) mechanisms. The purpose of this study was to assess the importance and the level of activation of non-homologous end joining (NHEJ) and homologous recombination (HR) DNA repair pathways in three cell lines, CCRF-CEM and MOLT-4 derived from T lymphocytes and SUP-B15 derived from B lymphocytes following treatment with chemotherapy agent daunorubicin.

**Results:**

The Gamma histone H2AX (γH2AX) assay was used assess the effects of DNA-PK inhibitor NU7026 and RAD51 inhibitor RI-2 on repair of DNA-DSB following treatment with daunorubicin. In all cell lines, the NHEJ DNA repair pathway appeared more rapid and efficient. MOLT-4 and CCFR-CEM cells utilised both NHEJ and HR pathways for DNA-DSB repair. Whereas, SUP-B15 cells utilised only NHEJ for DSB repair, suggestive of a deficiency in HR repair pathways.

## Introduction

DNA damage takes many forms including base deletion/addition/substitution, DNA adducts, single strand DNA breaks and the most lethal of all DNA double strand breaks (DNA-DSB). DNA-DSB are so lethal that one unrepaired DSB results in cell death. Hence, detection and repair of DNA-DSB is vital for cell survival. The presence of DNA-DSB triggers activation of Mre11, Rad50 and Nbs1 (MRN) complex and initiates downstream regulator proteins that facilitate DNA-DSB repair and death responses [1]. Major pathways involved in DNA-DSB repair processes include classical non-homologous end-joining (NHEJ), homologous recombination (HR) and the less understood alternative non-homologous end-joining (alt-NHEJ).

NHEJ forms the major DNA-DSB repair pathway in mammalian cells. The presence of DNA-DSB initiates activation and binding of the heterodimer complex Ku80 (Ku70 and Ku86). Ku heterodimer encircles the DNA to form an association with the DNA backbone and binding with blunt DNA end overhangs [2]. Ku80 binding to DNA-DSB recruits the catalytic subunit of DNA-dependent protein kinase (DNA-PK). DNA-PKs are members of phosphatidylinositol 3-kinase-related kinase (PIKK) family [3]. After recruiting DNA-PK to DNA-DSB, Ku turns one helical turn exposing DNA-PK to the base pair region at the DNA termini [1]. Once two broken ends of DNA-DSB bind with the Ku70/80-DNA-PK various processing factors stimulate the end processing of the non-ligatable DNA ends before DSB ends are appropriately ligated together to complete DNA-DSB repair by NHEJ [1]. Ku70 and Mre11 interact to form a complex of Ku-MRN which targets DNA-DSB together with the activation of ataxia telangiectasia mutated (ATM) and DNA-PK [4]. If NHEJ fails, the MRN complex is positioned to promote end joining by either HR or alt-NHEJ, each of which requires MRN to facilitate initial end resection. Activation of ATM by DNA-DSB induced by chemotherapy recruits breast cancer type 1 susceptibility protein (BRCA1), together with various other associated protein complexes such as BRCA2 and Rad51 to initiate DNA repair via HR [5]. HR functions with slower kinetics that rely on homologous template matching to repair the DNA-DSB and restore the DNA sequence at the break site and is restricted to S and G2 cell cycle phases [6].

Chemotherapeutic agents such as the topoisomerase II poisons (e.g. daunorubicin, doxorubicin, etoposide) are potentiated by NU7026 [7–10], a reversible inhibitor of DNA-PK involved in NHEJ [11]. In leukaemia cells NU7026 potentiates the effects of topoisomerase II poisons and results in a G2/M cell cycle arrest [10]. RI-2 also sensitises a broad range of cells to chemotherapy treatments. RI-2 is a Rad51 inhibitor that reversibly inhibits BRCA1, thereby leading to inhibition of HR in human cancer cells [12, 13].

Factors influencing choice between NHEJ and HR are still unclear as many factors influence these processes including MRN complex, cell cycle checkpoints, and DNA-DSB effector proteins [14]. The aim of this study was to assess the importance of DNA damage response pathways in acute lymphoblastic leukaemia cancer (ALL) cell lines following treatment with daunorubicin. The NHEJ and HR pathways were specifically inhibited with NU7026 or RI-2, alone and in combination following treatment with daunorubicin. The dynamic DNA repair processes were assessed over the next 24 h.

## Main text

### Methods

Cell lines used were CCRF-CEM and MOLT-4 (T-lymphoblastic leukaemia derivatives) and SUP-B15 (B-lymphoblastic leukaemia derivative) obtained from American Type Cell Culture Collection (ATCC) as previously described [15]. Daunorubicin, NU7026 and RI-2 were purchased from Sigma-Aldrich (NSW, Australia), prepared into stock solutions of 5 mM, 3 µM and 10 µM in dimethyl sulfoxide, respectively and aliquots were stored at ‒ 20 °C. Cells were plated onto 6-well plates at a seeding density of 1 × 10^6^ cells per well. Cells were treated with 10 μM daunorubicin and incubated for 4 h at 37 °C under 5% CO_2_, followed by recovery period of 4 h, 12 h or 24 h with or without 9 nM NU7026 or 100 nM RI-2. Treatment media was removed by centrifugation (200 g, 5 min), replaced with media only for further 4 h, 12 h or 24 h incubation. After recovery, cells were washed with PBS, fixed in 500 μL of 70% ethanol and processed as previously described [15].

## Results

**Fig. 1:**
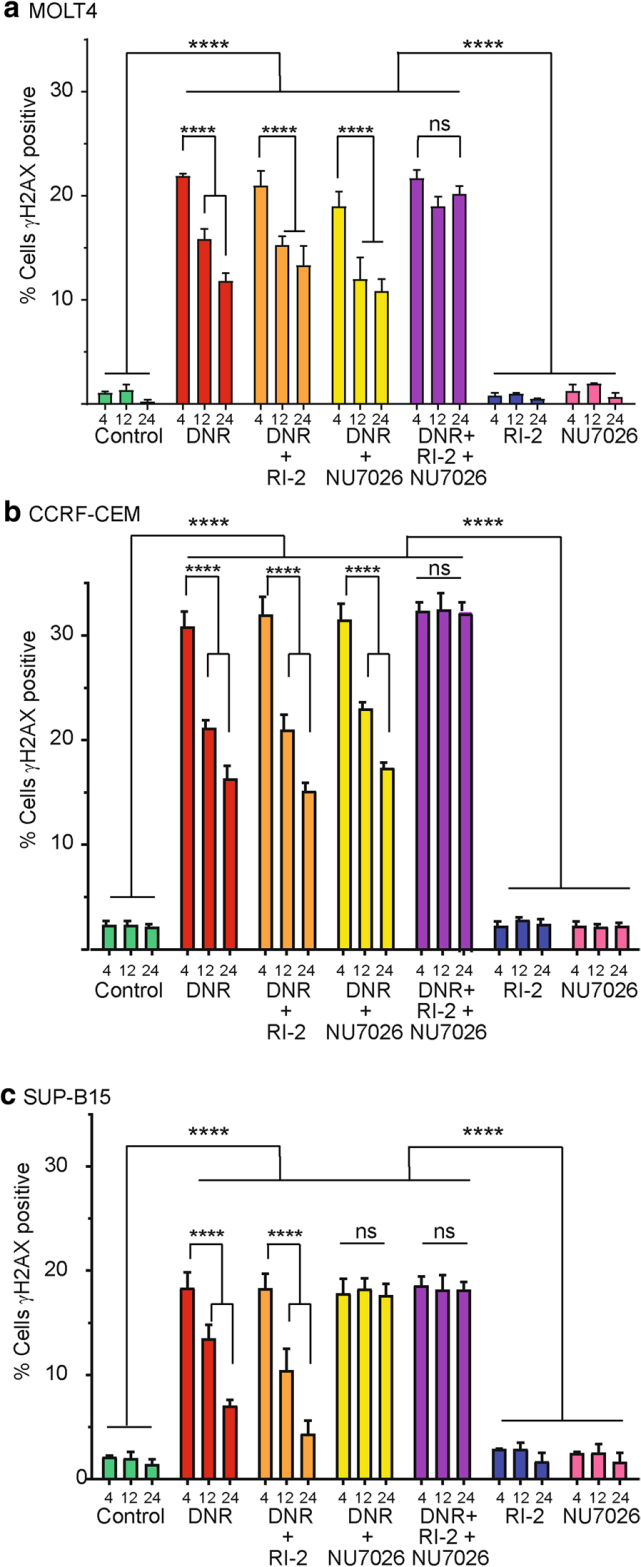
Measurement of DNA-DSB by γH2AX fluorescence for the different cell lines following daunorubicin (DNR) treatment. **a** MOLT-4 cells; **b** CCRF-CEM cells; **c** SUP-B15 cells. Cells were exposed to 9 nM NU7026 or 100 nM RI-2 alone or in combination with 10 μM DNR followed by 4 h, 12 h and 24 h in recovery media and the control treatment contained the vehicle dimethyl sulfoxide. Median intensities from histograms of the flow cytometry raw data were used to plot the bar diagrams (mean of total of six replicates ± SD, from three independent experiments). Statistical significance (****P < 0.0001; ns not significant P > 0.05) was determined by two-way ANOVA followed by Tukey’s multi-comparisons test

DNA repair following daunorubicin treatment was assessed by monitoring the disappearance of γH2AX over time (Fig. 1). The presence of DNA-DSB activates ATM and DNA-PK, which in turn phosphorylate H2AX at DSB sites transforming it into γH2AX [16]. Quantifying the γH2AX foci gives a measure of DNA-DSB and as these are repaired, γH2AX levels decrease, giving an indication of DNA repair.

The two T-lymphoblastic cell lines, MOLT-4 and CCRF-CEM (Fig. 1a, b), treated with daunorubicin alone showed increased DNA-DSB at 4 h and this declined after 12 h and then further after 24 h recovery indicating DNA repair. Similar events were seen in both daunorubicin plus RI-2 (BRCA1 inhibitor) and daunorubicin plus NU7026 (DNA-PK inhibitor) groups. However, when both pathways were simultaneously blocked in daunorubicin treated MOLT-4 and CCRF-CEM cells with both RI-2 and NU7026 inhibitors, high levels of DNA-DSB were seen at all recovery times indicating incomplete DNA repair.

When B-lymphoblastic derived SUP-B15 cells were treated with daunorubicin alone, there was a significant increase of DNA-DSB at 4 h which declined after 12 h and then further after 24 h of recovery media indicating DNA repair is occurring (Fig. 1c). Inhibition of the HR pathway with RI-2 plus daunorubicin resulted in a comparable level of γH2AX expression after 4 h, 12 h and 24 h in recovery. When the NHEJ pathway of repair was inhibited using NU7026 in combination with daunorubicin, the initial DNA-DSB formation was observed with no subsequent change in γH2AX expression, suggesting no DNA-DSB repair was occurring. Similarly, there was a significant increase in levels of DNA-DSB after 4 h when cells were treated with both NU7026 and RI-2 combined with daunorubicin, but no subsequent change in γH2AX expression, indicative of a lack of DNA-DSB repair.

To check the effects of NU7027 and RI-2 in the absence of daunorubicin, cells were exposed to either NU7026 or RI-2 alone. There was no significant increase in γH2AX expression compared to untreated cells (control) for each cell type (Fig. 1).

## Discussion

This study was designed to investigate mechanisms of DNA-DSB repair employed by ALL cell lines. We previously showed that DNA-DSB repair following daunorubicin treatment was lower in B-lymphocyte derived cells than in T-lymphocyte derived cells [15]. Here we show that DNA-DSB formation increases 4 h after exposure to daunorubicin and this is followed by a significant decrease in DNA-DSB during the recovery period, indicating DNA repair is occurring. Different cell lines show different background levels of γH2AX [17]. Therefore, a control group which contains untreated cells was included in the assay for each of the cell lines showing the background γH2AX intensity. DNA-PK, ATM and ATR contribute to activation of H2AX depending on a number of factors such as the cell cycle stage, type of DNA-DSB and the relative expression levels of individual PIKKs, which are not yet fully understood [18–20]. Therefore, known specific inhibitors of DNA-PK (NU7026; [7–11]) and BRCA1 (RI-2; [12, 13]) were included to analyse the relative roles of these proteins in DNA-DSB repair. Residual γH2AX after a recovery period can be used to quantify the ability of cells to repair their DNA-DSB after damage. To understand whether the effect observed was on DNA damage induction alone and/or repair, we examined the DNA repair kinetics in MOLT-4, SUP-B15 and CCRF-CEM cell lines after 4 h, 12 h and 24 h recovery periods following an initial exposure to daunorubicin with or without inhibitors.

Daunorubicin induced DNA-DSB in all investigated cells and this DNA damage was repaired but not completely within the 24 h timeframe. Addition of both NU7026 and RI-2 following daunorubicin treatment completely abolished any DNA-DSB repair, confirming that NHEJ and HR are the two important DNA-DSB repair pathways for these cells (Fig. 1). The T-lymphoblastic cell lines (MOLT-4 and CCRF-CEM; Fig. 1a, b) also exhibited DNA-DSB repair in RI-2 and NU7026 treated groups. RI-2 blocks HR DNA repair by inhibiting BRCA1, whilst NU7026 blocks NHEJ by inhibiting DNA-PK. Together, these data suggest that MOLT-4 and CCRF-CEM cell lines induced both HR and NHEJ DNA repair pathways and that neither pathway was solely relied on. Conversely, B-lymphoblastic SUP-B15 cells (Fig. 1c) treated with daunorubicin alone or daunorubicin and RI-2 (BRCA1 inhibitor) showed an increase level of DNA-DSB at 4 h that declined after 12 h and further after 24 h recovery indicating DNA repair. SUP-B15 cells treated with daunorubicin in combination with NU7026, retained high levels of DNA-DSB indicating incomplete repair. Therefore no alternative pathway occurred, suggesting that DNA-DSB repair in SUP-B15 is induced through NHEJ DNA repair only, possibly due to a defect in HR in these cells.

The NHEJ DNA repair pathway is efficient and quicker than HR pathway and this is consistent with other studies [21]. Two main factors that determine the speed of the DNA-DSB repair are heterochromatin (HC) complexity and DNA damage complexity [6, 22]. These factors consequently control the HR or NHEJ pathways utilised for DNA repair and slowly repaired DNA-DSB is a consequence of either damage or chromatin complexity preferentially undergoing DNA-DSB end resection [22]. NHEJ is the pathway of first choice as DNA-PK binds quickly to all DNA-DSB and this is attributed to Ku’s strong DNA-DSB -binding capacity or it is high occurrence [22]. If consequent steps of NHEJ progress well, then the DNA-DSB is repaired rapidly and sufficiently by NHEJ. When speedy re-joining cannot be achieved due to chromatin complexity or DNA damage complexity, then DNA-DSB end resection and HR occurs in G2 cell cycle phase, although NHEJ makes an additional try for DNA repair if resection cannot succeed [6]. Ku binding is also dynamic at DNA-DSB ends and it may be that Ku/DNA-PK is bound dynamically and release at the DNA end which allows a competition with resection [23].

In SUP-B15 cells, DNA repair is lower compared to CCRF-CEM and MOLT-4 cells where the γH2AX is significantly elevated after 4 h and then further decreases at 12 h and 24 h. This may because these cells utilised both NHEJ and HR repair pathways, while SUP-B15 cells utilised only NHEJ as DNA-DSB repair pathway. NHEJ repair pathway is more rapid and efficient than HR in most cells. The hierarchy of this regulatory network is still incompletely understood, though the balance between HR and NHEJ is intensely regulated. More comprehensive information of the precise network of cellular responses to DNA damaging treatments is required for development of targeted therapies using DNA damage response defects.

## Limitations

Limitations of this study include use of cell lines rather than primary cells obtained from cancer patients. However, two types of cells were chosen to partly overcome this limitation and these cells were all susceptible to daunorubicin treatment. Only one concentration of daunorubicin was used but this has previously been shown to be effective in these cell lines in addition to Jurkat T-lymphoma and HL-60 promyelocytic cell lines [15, 24]. Other topoisomerase poisons such as etoposide may confirm the favored DNA-DSB mechanisms suggested in this study. A surrogate measure of DNA repair was used by monitoring the disappearance of γH2AX over time and this should be confirmed with a direct measure of DNA repair efficiency such as neutral comet assays. Furthermore, activation of ATM in all cell types should be confirmed by immunoblot analysis. Another limitation is the lack of further investigation into potential proteins inactivated in the HR repair pathway in SUP-B15 cells. It is possible that BRCA1 or Rad51 are defective in these cells or other proteins. Mutations in BRCA1 results in high risk of breast and ovarian cancers [25]. BRCA1/BRCA2 deficient human and murine cells are sensitive to agents that damage DNA resulting in high production of DNA-DSB and impairment of cell cycle checkpoints. BRCA1 interacts with partner and localizer of the BRCA2 (PALB2) and BRCA2 at the site of DNA damage. Both PALB2 and BRCA2 facilitate RAD51-ssDNA filament formation [26]. BRCA1 dysfunction disturbs PALB2 and BRCA2 stability and so eliminates RAD51 localisation at DNA damage sites and revokes HR repair [27]. The continued accumulation of DNA-DSB may be due to a deficiency in BRCA1, resulting in SUP-B15 cells depending on the NHEJ DNA repair pathway but this remains to be shown by immunoblot analysis demonstrating a lack of BRCA1 activity for instance.

## Data Availability

All data presented or analyzed in this study are included in this article.

## Abbreviations

ALL: acute lymphoblastic leukemia
ATM: ataxia–telangiectasia mutated
BRCA1: breast cancer type 1 susceptibility protein
BRCA2: breast cancer type 2 susceptibility protein
DNA-DSB: dNA double strand breaks
γH2AX: gamma histone H2AX
HR: homologous recombination
NHEJ: non-homologous end joining
PALB2: partner and localizer of the BRCA2
